# Concurrent presentation of IgG4-related tubulointerstitial nephritis and ANCA MPO crescentic glomerulonephritis 

**DOI:** 10.5414/CNCS110852

**Published:** 2022-07-04

**Authors:** Henry H.L. Wu, Claire C.Y. Wang, Alexander Woywodt, Arvind Ponnusamy

**Affiliations:** 1 Department of Renal Medicine, Lancashire Teaching Hospitals NHS Foundation Trust, Royal Preston Hospital, Preston, and; 2 Faculty of Biology, Medicine and Health, The University of Manchester, Manchester, UK

**Keywords:** IgG4, tubulointerstitial nephritis, ANCA, MPO, crescentic glomerulonephritis

## Abstract

Concurrent IgG4-related tubulointerstitial nephritis and anti-neutrophil cytoplasmic antibodies (ANCA) myeloperoxidase (MPO) crescentic glomerulonephritis is an uncommon scenario, and the link between the two conditions, if any, is incompletely understood. We report the case of a 58-year-old woman who presented with a 2-month history of malaise and joint pain and was found to have acute kidney injury and hemato-proteinuria. Initial immunological tests revealed positive anti-neutrophil cytoplasmic antibodies with a peri-nuclear pattern (pANCA). An enzyme-linked immunoassay (ELISA) for anti-MPO antibodies was also positive, leading to a tentative diagnosis of ANCA-associated small vessel vasculitis with renal involvement. Steroid treatment was commenced, and an urgent kidney biopsy was performed. This showed crescentic glomerulonephritis, but also demonstrated concurrent tubulointerstitial nephritis with a dominance of IgG4-producing plasma cells. Serum IgG4 levels were also elevated. The patient was initially treated with intravenous cyclophosphamide and steroids and then switched to rituximab. When last seen, she was well after 1 dose of rituximab, with kidney function, inflammatory parameters, and serum IgG4 levels returning to normal levels. The concurrent presentation of ANCA-associated vasculitis and IgG4 renal disease is rare with only few cases reported in the literature. More work is needed to understand pathophysiology, outcomes, and management options for this complex scenario.

## Introduction 

Immunoglobulin G4 (IgG4)-related disease is a recently recognized syndrome which affects multiple organs through inflammation, fibrosis, or both [1]. The concept of IgG4-related disease was initially described in 2001 by Hamano et al. [2], who reported raised serum IgG4 levels in a subgroup of patients diagnosed with autoimmune pancreatitis. Since then, IgG4-related disease has been observed in the pancreas and biliary tract, salivary glands, lung, aorta and retroperitoneum, endocrine glands, and the kidney [3, 4, 5, 6, 7]. The commonest form of kidney involvement in IgG4-related disease is tubulointerstitial nephritis with both acute and chronic disease manifestations [7]. IgG4-related membranous nephropathy is also seen [7]. Concurrent IgG4-related disease and anti-neutrophil cytoplasmic antibody (ANCA)-associated vasculitis is very uncommon, and a distinct new syndrome has been proposed [8]. We report a case of concurrent IgG4-related tubulointerstitial nephritis and ANCA MPO crescentic glomerulonephritis. We describe our case and provide a brief review of the literature in terms of pathophysiology, diagnostic recommendations, and management options based on current evidence. 

## Case report 

A 58-year-old previously healthy woman without significant past medical history presented feeling generally unwell and fatigued over the preceding 2 months. The patient did not usually take any regular medications. She reported a recent history of intermittent joint pains in her hands, which had become more severe over the past week despite a short course of non-steroidal anti-inflammatory medications (naproxen). Active urinary sediment had been noted elsewhere in the preceding 1-month period prior to attendance to nephrology clinic when the patient visited the emergency department of her local district general hospital, though urine microscopic analysis was otherwise unremarkable. The patient did not report any ocular or nasal symptoms, rash, or hemoptysis. She was hypertensive with a blood pressure of 200/118, while the remainder of the clinical examination was unremarkable. Urine microscopic analysis was positive for protein (0.8 g proteinuria) and blood (< 50 red blood cells/high-power field). Blood tests showed evidence of acute kidney injury with serum creatinine 227 µmol/L (baseline serum creatinine 67 µmol/L) and moderately raised inflammatory markers, with C-reactive protein being 102 mg/L, and anemia (hemoglobin 85 g/L (normal, 110 – 160 g/L)). Liver function tests and coagulation studies were all normal. Immunology screening identified serum positivity to ANCA and abnormal levels of the myeloperoxidase (MPO) enzyme-linked immunoassay (ELISA), with MPO ELISA titer being 55 IU/mL (normal range < 20 IU/mL). Immunology tests including complement C3 and C4, antinuclear antibodies, anti-glomerular basement membrane antibodies, free light chains, were all unremarkable. Chest X-ray and computed tomography of the chest and abdomen were not suggestive of any systemic pathology, including lymphadenopathy. 

**Figure 1.**
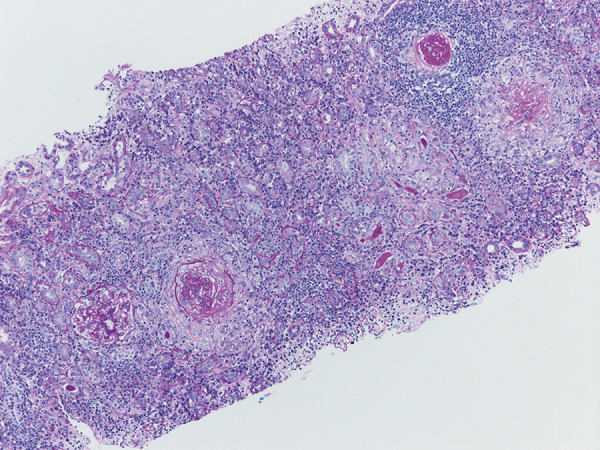
Crescentic glomerulonephritis with hematoxylin and eosin stain (at × 20 magnification).

**Figure 2.**
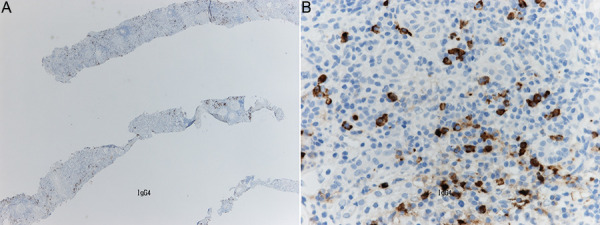
A: Tubulointerstitial inflammation with a predominance of IgG4-producing plasma cells in the kidney parenchyma (at × 2 magnification). B: High-powered view with immune deposit staining confirming the presence of IgG4 deposits (at × 40 magnification).

We made a tentative diagnosis of MPO-ANCA-associated small vessel vasculitis with renal involvement. An urgent renal biopsy was scheduled. The ANCA MPO titer was only mildly raised above the normal reference range, and the patient was well with stable kidney function, hence oral steroid therapy (prednisolone 30 mg once daily) was initiated 3 days prior to kidney biopsy. The kidney biopsy showed cellular and fibrous crescents with negative immunohistochemistry, in keeping with pauci-immune glomerulonephritis (Figure 1). There was moderate tubulointerstitial fibrosis. Surprisingly, significant tubulointerstitial inflammation was also seen with a predominance of plasma cells (Figure 2A). These were later confirmed to be IgG4-producing plasma cells on immunohistochemistry, with the presence of IgG4 deposits confirmed following immune deposit staining, and the infiltration of 10 IgG4 cells per high-power field in keeping with IgG4-related disease (Figure 2B). Further serum samples were sent to determine serum IgG4 levels, which were found to be raised at 1.94 g/L (reference range is ≤ 1.35 g/L). 

The patient was commenced on cyclophosphamide 1 g intravenously with further doses of cyclophosphamide scheduled, whilst her daily prednisolone dose continued. The patient’s blood pressure control was also optimized during this time with amlodipine 5 mg once daily, and she was commenced on atorvastatin 20 mg daily. Oral co-trimoxazole and fluconazole were started for prophylaxis of infection. The patient was discharged in good health. We discussed further immunosuppression for this unusual presentation and decided to switch her immunosuppressive regime to rituximab, due to its presumed ability to effectively treat both IgG4-related disease and ANCA-associated vasculitis with a milder side-effect profile compared to cyclophosphamide. Rituximab was initiated with single-dose intravenous methylprednisolone 250 mg, and oral prednisolone was reduced to 15 mg daily. When last seen in clinic after 1 dose of rituximab, the patient appeared well with her kidney and inflammatory parameters much improved (serum creatinine 159 µmol/L and C-reactive protein < 1.0 mg/L), and serum IgG4 levels falling back to within normal ranges (0.52 g/L). No active urinary sediment was detected. The patient also reported settling of her joint pain symptoms in this clinic appointment following the multiple immunosuppressive treatments administered. She was scheduled for her second dose of rituximab treatment 2 weeks following this appointment. 

## Discussion 

Concurrent subacute presentation of IgG4-related kidney disease with ANCA-associated MPO crescentic glomerulonephritis is uncommon, with cases reporting these simultaneously presenting diagnoses only surfacing over recent years (Table 1) [9, 10, 11, 12, 13, 14]. In 2017, Su et al. [10] reported the first case of concurrent IgG4-related tubulointerstitial nephritis with ANCA-associated MPO crescentic glomerulonephritis in a 42-year-old Chinese man. The patient had been diagnosed with IgG4-related autoimmune pancreatitis 1 year prior to the presentation with kidney disease. Reports of IgG4-related disease and ANCA MPO vasculitis without kidney involvement have also been noted in cases where patients initially presented with otitis media, rhinosinusitis, hypophysitis, and pachymeningitis [15, 16, 17]. 

Whether or not there is a causal link between IgG4-related disease and ANCA-associated vasculitis is currently unclear. It is worthwhile considering the role of different immunoglobulin subclasses in ANCA-associated vasculitis. ANCA is predominantly of the IgG isotype, and IgG1, IgG3, and IgG4 are mostly representative of the IgG isotype [18]. Compared to IgG1 and IgG3, IgG4 is recognized as a comparatively less pathogenic antibody in the pathophysiology of primary ANCA-associated systemic vasculitis [16]. Experimental studies suggest that the IgG4 subclass of ANCA proteinase 3 ligates FcγRIIa/IIIb receptors and activates neutrophils [19]. However, robust experimental data linking IgG4 and ANCA MPO are lacking. Whether IgG4-producing plasma cells produce MPO antibodies, also requires further study. An observational study of 92 ANCA-associated vasculitis patients conducted by Ma et al. [18] identified 10 patients who had concurrent IgG4 disease. They demonstrated that serum IgG4 levels of ANCA MPO was much higher in patients with IgG4-related disease and ANCA-associated vasculitis than in patients with only ANCA-associated vasculitis. Serum IgG1, IgG2, and IgG3 levels were comparable between the two groups. Furthermore, immunofluorescence testing revealed co-localization of MPO and IgG4 in the mesangial cells of the IgG4-related disease and ANCA-associated vasculitis group. The authors speculated that given the diffuse infiltration of IgG4-producing plasma cells in the kidneys, IgG4-producing plasma cells may generate IgG4 antibodies, leading to neutrophil activation [18]. 

Histological identification of IgG4-producing plasma cells with concurrent crescentic glomerulonephritis in the presence of positive ANCA ELISA testing led to the diagnosis in the case presented here [20]. Serum IgG4 is a sensitive marker for IgG4-related disease, but currently considered to lack diagnostic specificity [21]. It is also worth considering the fact that both IgG4 immunohistochemistry in renal biopsies and serum IgG4 measurements are not easily accessible worldwide. The latter is certainly not currently part of routine blood tests in patients with unexplained renal impairment. Of note, elevated serum IgG4 levels are somewhat non-specific and also seen in chronic inflammatory conditions (e.g., inflammatory bowel disease) and malignancies (e.g., lymphoma) [22, 23]. It is therefore possible that some cases of this unusual presentation are not diagnosed and reported. Clinicians should be aware of the association, and use of IgG4 immunohistochemistry in renal biopsies and measurement of serum IgG4 should be employed in unusual cases of ANCA-associated vasculitis, for example when there is tubulointerstitial nephritis or fibrotic extra-renal disease. In summary, a high degree of suspicion is required to validate this unusual combination of diagnoses. 

The evidence for treatment of this unusual presentation is weak simply due to the rarity of this presentation, and larger studies seem unlikely at present. Anecdotal reports consider cyclophosphamide combined with steroid therapy as a potentially effective treatment option for concurrent IgG4-related kidney disease and ANCA-associated vasculitis [9, 24]. Rituximab use has been described in the treatment of IgG4-related kidney disease due to its ability to deplete B cells and having a better side-effect profile compared to cyclophosphamide combined with steroid therapy [25, 26, 27]. It is tempting to think of rituximab as a potentially ideal drug in treating the concurrent presentation of IgG4 disease and ANCA-associated vasculitis, but this assumption will require further exploration [28, 29]. 

## Conclusion 

Our report of concurrent IgG4-related tubulointerstitial nephritis and ANCA MPO crescentic glomerulonephritis adds another case to the existing literature of this uncommon presentation. The etiology of IgG4 disease remains unclear at present, and whether or not the concurrent presentation with ANCA-associated vasculitis constitutes a distinct syndrome is therefore difficult to say. Improved understanding of the pathophysiology in IgG4 disease may help to delineate a causal link, if any, between the two. Further reports of this unusual combined presentation in patients from diverse ancestry are needed to aid our understanding of this condition. We suggest increased vigilance in patients presenting with unusual features of ANCA-associated vasculitis as well as a low threshold for performing IgG4 immunohistochemistry in renal biopsies and measurement of serum IgG4. Further case reports may also help our understanding of treatment options for this uncommon condition. 

## Acknowledgment 

Informed Consent was obtained from the patient. The authors would like to acknowledge Dr. Beena Nair (Consultant Histopathologist) for providing the case images. 

## Funding 

There was no external financial support for this article. 

## Conflict of interest 

The authors declare there is no conflict of interest and have no disclosures to be made for this article. 

**Table 1.** Summary of published cases of concurrent presentation of IgG4-related tubulointerstitial nephritis and ANCA MPO crescentic glomerulonephritis

Author, country of publication, journal and year of Publication	Patient gender, and age	Presenting symptoms and significant past medical history	Key serum investigation results	Kidney biopsy findings	Treatment and outcome
Tamai et al. [9] Japan Allergy, Asthma and Clinical Immunology 2011	Male 72 years	General lymphadenopathy; recurrent HSP	Increased serum IgE and IgG; IgG4; serum protein electrophoresis revealed polyclonal hypergammaglobulinemia; C3, C4, and total complement hemolytic activity all reduced; ANCA MPO 22 EU (normal, < 10 EU)	Light Microscopy Interstitial inflammation with lymphocytes and plasma cells, and concurrent segmental glomerulonephritis	Oral prednisolone
					Lymphadenopathy has decreased though there was continued hematuria and proteinuria
				Immunohistochemistry Positive for IgA and IgG in the mesangium; IgG4 antibody staining revealed many positive plasma cells in the interstitium	
				Electron Microscopy Numerous electron-dense deposits in the mesangium	
Su et al. [10] PRC Medicine 2017	Male 42 years	Epigastric pain and AKI; previous presentation of acute pancreatitis one year ago	Serum creatinine is 157 µmol/L; serum IgG4 was 1.84 g/L; pANCA positive and ANCA MPO titer levels > 200 IU/mL	Light Microscopy Severely disrupted glomerular capillary loops with cellular crescents, rupture of Bowman capsule, peri-glomerular granulomata formation Diffuse lymphocyte and plasma cell infiltration evident in tubulointerstitium	Pulsed methylprednisolone, oral prednisolone, Intravenous cyclophosphamide
					Serum creatinine and IgG levels decreased; chronic glomerular crescents with “storiform” interstitial fibrosis in second kidney biopsy
				Immunohistochemistry Most of the infiltrate was CD138-positive cells More than 40% were IgG4-positive plasma cells Enriched IgG4-positive plasma cell interstitial infiltration	
Galante et al. [11] U.K. BMC Nephrol 2020	Male, 51 years	Frank hematuria, proteinuria and severe AKI; ex-smoker with Graves Disease’ and asthma; recommenced on PTU treatment 2 weeks prior to presentation	Serum creatinine on admission was 568 μmol/L (116 μmol/L one year ago); C-reactive protein was 100 mg/L; Positive titers for anti-GBM (94 IU/mL), p and cANCA (1 : 80 titer), anti-PR3 (76.9 IU/mL) and anti-MPO (28.8 IU/mL); serum IgG4 normal	Light Microscopy Cellular crescents with little or no organization in 50% of glomeruli Segmental necrosis and tubular atrophy Widespread lymphoplasmacytic infiltrate, associated with tubulitis	Pulsed intravenous methylprednisolone before biopsy; PTU stopped, low-dose carbimazole replacing PTU; required plasma exchange; discharged on oral prednisolone
				Immunohistochemistry Mesangial positivity for IgA, C3 and lambda Lymphoplasmacytic infiltrate noted, IgG4 staining demonstrated > 30 IgG4 positive plasma cells per high-power field	
					IgG4 and ANCA titers maintained within normal range, serum creatinine stabilized
				Electron Microscopy Paramesangial and subendothelial electron-dense deposits associated with endocapillary hypercellularity typical of IgA nephropathy	
Li et al. [12] PRC Clin Chim Acta 2020	17 patients	12 patients presented with AKI; 14 had multi-organ pathology on presentation	ANCA positivity; abnormal levels of the MPO ELISA and raised serum IgG4 for all 17 patients	Features of tubulointerstitial nephritis and crescentic glomerulonephritis identified in all 17 patients	2 patients received oral prednisolone only – 1 had improved kidney function, the other became HD dependent
					14 patients received cyclophosphamide combined with oral prednisolone – 11 had improved kidney function, 3 had treatment resistance of which 2 required HD
					1 patient received rituximab with steroids and had improved kidney function
Feng et al. [13] PRC Medicine 2020	Male, 72 years	Intermittent fever, fatigue and full-body discomfort; had frank hematuria and proteinuria	Serum creatinine is 233.9 µmol/L; Serum IgG4 was 7.23 g/L; pANCA positive and ANCA MPO titer levels is 203.45 IU/mL	Light Microscopy Necrotizing crescentic glomerulonephritis and plasma cell infiltrates in the renal interstitium	Methylprednisolone and Cyclophosphamide; adalat for hypertension and erythropoietin agents for anemia.
				Immunohistochemistry Most of the IgG-positive infiltrating inflammatory cells were IgG4 immunoreactive, accounting for over 40% of the IgG-positive cells. Diffuse granular mesangial deposition of C3	
					Clinical improvement with no disease recurrence; improvement in biochemical parameters
				Electron Microscopy Subepithelial intramembranous and mesangial deposits	
Lu et al. [14] PRC Ann Clin Lab Sci. 2021	Male, 54 years	Sicca symptoms; past medical history of Sjögren’s syndrome for 20 years.	Progressive renal dysfunction; positive anti-Ro/SS-A, anti-La/SS-B antibodies, MPO-ANCA; significant increase of serum IgG4 level	Light Microscopy Necrotizing glomerulonephritis with crescents with severe tubulointerstitial nephritis	Rituximab combined with steroids
				Immunohistochemistry Extensive infiltration of IgG4-positive plasma cells	Clinical improvement and improvement in biochemical parameters

ANCA = antineutrophil cytoplasmic antibody; AKI = acute kidney injury; C3 = complement 3; CRP = C-reactive protein; ELISA = enzyme-linked immunoassay; ESR = erythrocyte sedimentation rate; GBM = glomerular basement membrane; HSP = Henoch Schonlein purpura; HD = hemodialysis; IgA = immunoglobulin A; IgE = immunoglobulin E; IgG = immunoglobulin G; IgM = immunoglobulin M; MPO = myeloperoxidase; PRC = People’s Republic of China; PTU = propylthiouracil.
